# Supplementary material for: Deletion of wfs1 Impairs Oligodendrocyte Precursor Cells Dorsal Distribution and Myelination Through the wfs1-hmgcs1 Axis in Zebrafish
Source: Biology (Basel). 2026 Aug 16;15(16):1402. doi: 10.3390/biology15161402 (PMC13510151; doi:10.3390/biology15161402)
Supplement: Supplementary file 1 [file biology-15-01402-s001.zip › File S1-Transcriptome sequencing data and differentially expressed genes (DEGs) in wfs1-- zebrafish at 5 dpf/2.KEGG/wfs1KOvsWT/ALL/src/dre03460.html]

dre03460


- dre:402936

- Up regulated genes

402936\_1(1.9435116777903)

- Down regulated genes

402936(-3.19856244926314)

- dre:558923

- Up regulated genes

558923(6.88953216261849)

- Down regulated genes

558923\_1(-1.7046322185763)

- dre:794038

- Up regulated genes

794038(1.51724035153863)

- Down regulated genes

794038\_1(-5.39870962384233)

- dre:450081

- Up regulated genes

450081(1.15186882580158)

- dre:570043

- Up regulated genes

570043\_1(1.22985575688127)

- dre:100538153

- Up regulated genes

100538153(2.31850635345817)

- Down regulated genes

100538153\_1(-1.8662486543101)

- dre:406487

- Up regulated genes

406487(7.45084509342341)

Close
